# The Protective Effects of the Supercritical-Carbon Dioxide Fluid Extract of *Chrysanthemum indicum* against Lipopolysaccharide-Induced Acute Lung Injury in Mice via Modulating Toll-Like Receptor 4 Signaling Pathway

**DOI:** 10.1155/2014/246407

**Published:** 2014-08-25

**Authors:** Xiao-Li Wu, Xue-Xuan Feng, Chu-Wen Li, Xiao-Jun Zhang, Zhi-Wei Chen, Jian-Nan Chen, Xiao-Ping Lai, Sai-Xia Zhang, Yu-Cui Li, Zi-Ren Su

**Affiliations:** ^1^School of Chinese Materia Medica, Guangzhou University of Chinese Medicine, No. 232, Waihuandong Road, Guangzhou Higher Education Mega Center, Guangzhou 510006, China; ^2^Faculty of Health Science, University of Macau, Taipa 999078, Macau; ^3^State Key Laboratory of Quality Research in Chinese Medicine, Institute of Chinese Medical Sciences, University of Macau, Taipa 999078, Macau; ^4^Dongguan Mathematical Engineering Academy of Chinese Medicine, Guangzhou University of Chinese Medicine, Songshan Lake High-Tech Industrial Development Zone, Dongguan, Guangdong 523808, China

## Abstract

The supercritical-carbon dioxide fluid extract of *Chrysanthemum indicum* Linné. (CFE) has been demonstrated to be effective in suppressing inflammation. The aim of this study is to investigate the preventive action and underlying mechanisms of CFE on acute lung injury (ALI) induced by lipopolysaccharide (LPS) in mice. ALI was induced by intratracheal instillation of LPS into lung, and dexamethasone was used as a positive control. Results revealed that pretreatment with CFE abated LPS-induced lung histopathologic changes, reduced the wet/dry ratio and proinflammatory cytokines productions (TNF-*α*, IL-1*β*, and IL-6), inhibited inflammatory cells migrations and protein leakages, suppressed the levels of MPO and MDA, and upregulated the abilities of antioxidative enzymes (SOD, CAT, and GPx). Furthermore, the pretreatment with CFE downregulated the activations of NF-*κ*B and the expressions of TLR4/MyD88. These results suggested that CFE exerted potential protective effects against LPS-induced ALI in mice and was a potential therapeutic drug for ALI. Its mechanisms were at least partially associated with the modulations of TLR4 signaling pathways.

## 1. Introduction

Acute lung injury (ALI) and its severe form, acute respiratory distress syndrome (ARDS), are clinical problems induced by acute and excessive inflammatory responses to stimulus in the airspaces and lung parenchyma, involving alveolar-capillary membrane damage, neutrophils recruitment, vascular permeability increase, pulmonary edema, and respiratory failure [1–3]. ALI and ARDS are life-threatening problems, always leading to multiple organ dysfunction syndrome (MODS), with significant incidences and mortalities in critically ill patients [1, 2]. Although the pathologies of ALI have been clearly defined and several candidate therapy strategies have been clinically applied for ALI [3–5], there is still no effective therapy strategy but relatively noteworthy mortality rates [2, 6]. Therefore, to develop novel effective prevention and therapies is urgently needed.

Lipopolysaccharide (LPS), a major constituent of outer membranes of Gram-negative bacteria, has been defined to be a pivotal risky factor and prominent stimulus in the pathogenesis of ALI [3, 7]. It has been defined that LPS challenges induce neutrophil infiltrations, trigger acute inflammatory responses, and generate early lung pathological changes, resulting in the morbidity and development of ALI [3, 8, 9]. Thus intratracheal instillation of LPS to experimental animals, with clear clinical relations to the process of ALI but no systemic inflammation, is well-suited and reproducible for preliminarily pharmacological researches of novel drugs or other therapeutic agents [8–10]. Once entering into the host, LPS activates toll-like receptor 4 (TLR4), a main sensor of the TLR superfamily acting as transmembrane proteins and signal transduction molecules [5, 7, 11]. TLR4 can activate nuclear factor- (NF-)*κ*B protein through myeloid differentiation factor 88 (MyD88) pathway [5, 11]. Then the activated NF-*κ*B induces the productions of proinflammatory cytokines such as tumor necrosis factor- (TNF-)*α*, interleukin- (IL-) 1*β*, and IL-6 [11–13], resulting in recruitment of intravascular neutrophils into the alveolar space and lung parenchyma [14, 15], followed by protease release and reactive oxygen species (ROS) generation which is closely associated with lipid peroxidation aggravation (such as malondialdehyde, MDA) and antioxidant enzyme (such as superoxide dismutase, SOD; catalase, CAT; and glutathione peroxidase, GPx) activity decline [8, 14]. Hence, drugs focusing on downregulating the TLR4 signaling pathway and/or inhibiting its related inflammatory responses would provide potential therapeutic effects for ALI [5, 11].

*Chrysanthemum indicum* Linné (*C. indicum*), a traditional Chinese medicine, has long been used in treatments of several acute respiratory system diseases, including cold, cough, acute bronchitis, acute laryngitis, and acute pharyngitis, with high efficacy and low toxicity [16]. The supercritical-carbon dioxide fluid extract of* C. indicum* (CFE) has been widely applied as a fine material in many TCM preparations, for example,* C. indicum* granules and capsules [17, 18]. Most importantly, CFE was a key ingredient of a TCM recipe named Compound* C. indicum* Soft Capsule (also known as CPZ in previous studies), an anti-influenza drug whose effects were closely associated with anti-inflammatory activity [18]. CFE has also been demonstrated to possess strong anti-inflammatory effects, and the underlying mechanism was related to its upregulations of antioxidant enzymes and downregulations of NF-*κ*B and some proinflammatory cytokines, such as TNF-*α*, IL-1*β*, and IL-6 [17, 18].

Although CFE has shown anti-inflammatory benefits, its potential effects to protect against LPS-induced ALI still remained unclear. Therefore, in this present study, we aimed to evaluate the effect of CFE on LPS-induced ALI in mice. And for potential mechanisms elucidation, the expressions of TLR4/MyD88 and the phosphorylations of NF-*κ*B p65/I*κ*B*α* were also evaluated.

## 2. Materials and Methods

### 2.1. Drugs and Chemicals

Lipopolysaccharide (LPS) and dexamethasone (Dex) were purchased from Sigma Co., Ltd. (St. Louis, USA), and Xianju Pharmaceutical Co., Ltd. (Zhejiang, China), respectively. Phosphate buffered saline (PBS), sodium dodecyl sulphate polyacrylamide gel (SDS-PAGE), skimmed milk, Tween-20, and Tween-80 were purchased from Thermo-Fisher Sci. Co., Ltd. (MA, USA). Hexadecyltrimethylammonium bromide (HTAB) and* o*-dianisidine were purchased from TCI Co., Ltd. (Tokyo, Japan). All other chemicals were of the reagent grade.

### 2.2. Preparation of CFE

The supercritical fluid CO_2_ extract of* Chrysanthemum indicum* Linné (CFE) was prepared and provided by the Institute of New Drug Research & Development, Guangzhou University of Chinese Medicine (Lot. 20121104). In our previous chemical analysis of CFE, 35 compounds were identified by GC-MS, and 5 compounds were reconfirmed and quantified by HPLC-PAD (the brief chemical profile of CFE is listed in Table 1) [17]. For experiments in this paper, CFE samples were diluted by 0.5% Tween-80 into the appropriate dose.

### 2.3. Experimental Animals

Male Kunming (KM) mice (20–25 g) were purchased from Medical Laboratory Animal Center of Guangdong Province (Certificate number SCXK2008-0002, Guangdong Province, China). Animals were kept on 12-hour light/12-hour dark cycles under regular temperature (22 ± 2°C) and humidity (50 ± 10%) with standard diets and clean water* ad libitum*. All animals were sacrificed by lethal sodium pentobarbital injection. All experiments were conducted according to the National Institutes of Health Guide for the Care and Use of Laboratory Animals and approved by the Institutional Animal Care and Use Committee of Guangzhou University of Chinese Medicine.

### 2.4. Experimental Designs

#### 2.4.1. Positive Control Setting, Animal Groups, and LPS-Induced ALI

Dex has been frequently used as a positive control agent in various kinds of ALI models in experimental settings. With the definite and proved benefits and effects, Dex is applicable to be the positive control agent for screening and evaluating new therapeutic agents [19–21]. Therefore, Dex was selected as the positive control agent and a common dose of 5 mg/kg (p.o.) used in this study [19–21].

To assess mortality rate, 120 mice were randomly divided into 5 groups (*n* = 24), sham group, LPS group, and CFE (40, 80 and 120 mg/kg) groups. CFE groups were given CFE (40, 80, and 120 mg/kg, p.o.) while sham group and LPS group were given Tween-80, for 7 consecutive days. One hour after the last administration, all animals were anesthetized. And mice from LPS group and CFE groups were given a single intratracheal instillation of 20 mg/kg LPS (10 mg/mL, dilution with PBS; 20 *μ*L/10 g body weight) while mice from the sham group were given an equal volume of PBS. After operation, mice of all groups were monitored and the time when any animal died was recorded every 6 hours up to 120 hours. Then the mortality rate of each group within 120 hours was calculated and compared using the Kaplan Meier methods.

In the other experiments, mice were randomly divided into 6 groups (*n* = 30), sham group, LPS group, CFE (40, 80, and 120 mg/kg) groups, and Dex group (5 mg/kg). CFE groups and Dex group were given CFE (40, 80, and 120 mg/kg, p.o.) and Dex (5 mg/kg, p.o.) once per day for 7 consecutive days, respectively. During this period, sham group and LPS group were given equal volumes of Tween-80. One hour after the last administration, mice were anesthetized via intraperitoneally injecting pentobarbital sodium (30 mg/kg). After that, mice from LPS group, Dex group, and CFE groups were given a single intratracheal instillation of 5 mg/kg LPS (2.5 mg/mL, freshly diluted with PBS; 20 *μ*L/10 g body weight) while mice of sham group were given an equal volume of PBS. In this model, all animals survived for 24 hours after the intratracheal instillation of LPS at the dose of 5 mg/kg, which was optimized and repeatable, based on our preliminary experiments (data were not provided).

#### 2.4.2. Specimen Collections

24 hours after LPS instillation, 30 mice of each group were randomly divided into 3 parts, 10 mice per part. Part 1 was used for the bronchoalveolar lavage fluid (BALF) preparation and the lung wet/dry weight (W/D) ratio measurement. In brief, after anaesthetization, mouse was surgically exposed the trachea and clamped the right main bronchus. Then the left lung of each animal was lavaged for three times with a total volume of 1.5 mL of Hanks-Balanced-Salt solution using a venous indwelling needle. The BALF recovery rate was more than 90%. Then the mouse was sacrificed, and right lung was harvested for the W/D ratio measurement. Part 2 was used for the lung tissue preparation and the histopathologic evaluation. In brief, after the mouse was sacrificed, the left lung was taken, placed in appropriate amount of precold PBS immediately, and homogenized using a Tissue Lyser II high-throughput tissue homogenization system (Qiagen Co., Ltd., Hilden, Germany). Then the homogenate was centrifuged at 4°C. 100 *μ*L of the supernatant was used for protein measurement and the rest was immediately collected and stored at −80°C for further analysis of MPO, MDA, proinflammatory cytokines (TNF-*α*, IL-1*β*, and IL-6), and antioxidant enzymes (SOD, CAT, and GPx). At the same time, the right lung was harvested for the histopathologic examination. Part 3 was left for western-blot assay of TLR4, MyD88, NF-*κ*B p65, and I*κ*B*α*. In brief, mouse was sacrificed, and the lung tissues were harvested, frozen, and stored in liquid nitrogen immediately.

### 2.5. Measurement of Lung W/D Ratio

The lung W/D ratio was measured according to the previous study. In brief, the excised right lung was blotted dry and weighed to obtain the “wet” weight, afterwards kept in an oven at 80°C for 48 hours to obtain the “dry” weight. Then the W/D ratio was calculated by the “wet” weight to the “dry” weight.

### 2.6. Measurement of BALF Protein Contents and BALF Cell Counts

The BALF was centrifuged at 800 ×g for 10 min at 4°C, and the supernatant was collected for measurement of protein content. Measurements of protein contents of BALF were performed using a commercial BCA kit (Beyotime Institute of Biotechnology, Shanghai, China) and expressed as mg/mL BALF. Then sediment cells were resuspended in precold PBS and stained by a Wright-Giemsa kit (Nanjing Jiancheng Bioengineering Institute, Nanjing, China) for cytospin preparations. Counts of the total cells, neutrophils, and macrophages were then double-blindly performed via hemacytometry.

### 2.7. MPO Assay

The assay of MPO activity was performed via the HTAB method. Briefly, the samples were mixed with KPO_4_ buffer (50 mM, pH 6.0) with HTAB (0.5%). After reacting and incubating at 37°C for 15min, the enzyme was assayed by the activity in a H_2_O_2_/*o*-dianisidine buffer at 460 nm with a Multiskan GO microplate spectrophotometer (Thermo-Fisher Sci., Waltham, USA). Results were expressed as units/mg protein.

### 2.8. Histopathologic Examination

Biopsies of right lungs were collected, fixed in 4% paraformaldehyde solution, dehydrated, embedded with paraffin, and sectioned into 4 *μ*m. Tissue sections were stained with hematoxylin and eosin kit (H&E, Beyotime Institute of Biotechnology), examined, and photographed using TE2000-S Inverted Microscopes (Nikon Co., Ltd., Tokyo, Japan). According to previous reports, histologic changes including neutrophil infiltration, interstitial edema, congestion (or hemorrhage), and hyaline membrane formation were evaluated and the severity of each change was scored on a scale of 0 (normal) to 4 (severe) by a pathologist blinded to this study. Finally, the overall histologic injury was evaluated according to the sum-scores (0 as normal, 1 to 5 as minimal; 6 to 10 as mild; 11 to 15 as moderate; 16 to 20 as severe). Results were presented as the means of scores of microscopic areas of each group.

### 2.9. MDA, SOD, CAT, and GPx Assay

The MDA assay was carried out with commercial MDA assay kits (Beyotime Institute of Biotechnology) via the method of thiobarbituric acid reacting substance (TBARS). In Brie, reaction between MDA and TBARS was performed at 100°C, resulting in formation of a red complex TBARS, which could be recorded and measured at 532 nm. SOD, CAT, and GPx were measured using commercially available kits (Beyotime Institute of Biotechnology) according to the manufacturers' instructions, respectively. Briefly, xanthine and xanthine oxidase (XOD) generated superoxide radicals and reacted with 2-(4-iodophenyl)-3-(4-nitrophenol)-5-phenyltetrazolium chloride to form a red formazan dye, which could be measured at 532 nm. CAT was measured by the production of N-(4-antipyryl)-3-chloro-5-sulfonate-p-benzoquinonemonoimine, which would be detected at 520 nm. And GPx was measured by detecting contents of GRd and NADPH, which would be recorded at 340 nm.

### 2.10. ELISA Assay for TNF-*α*, IL-1*β*, and IL-6

TNF-*α*, IL-1*β*, and IL-6 were measured using commercially available ELISA kits (*e* Bioscience Co., Ltd., CA, USA). In brief, diluted standards or samples were added to 96-well plates precoated with affinity purified polyclonal antibodies specific for mouse TNF-*α*, IL-1*β*, and IL-6, respectively. Then wells were added with enzyme linked polyclonal antibodies and incubated at 37°C for 60 min, followed by final washes for 5 times. The intensities detected at 450 nm were measured after addition of substrate solutions for 15 min. Levels of TNF-*α*, IL-1*β*, and IL-6 were calculated according to standard curves.

### 2.11. Western-Blot Assay for TLR4, MyD88, and NF-*κ*B

Extractions of proteins from the lung tissues were performed with T-PER tissues proteins extractions reagent kits (Beyotime Institute of Biotechnology). Extractions of nuclear and cytoplasmic proteins from the lungs were performed with nuclear and cytoplasmic proteins extractions reagent kits (Beyotime Institute of Biotechnology). Protein contents were measured using BCA protein assay kits and equal amounts of protein were added in per well on 10% SDS PAGE. Then, proteins were separated and transferred into PVDF membranes by an Electrophoresis System (Bio-rad Co., Ltd., Hercules, USA). The resulting membranes were blocked with Tris-buffered-saline containing 0.05% Tween-20 (TBS-T), supplemented with 5% skimmed milk at room temperature for 2 hours, and followed by TBS-T washings. Then membranes were incubated with related specific primary antibodies anti-NF-*κ*B p65 antibody, anti-I*κ*B*α* antibody (Cell Signaling Technology Co., Ltd., MA, USA), anti-TLR4 antibody, and anti-MyD88 antibody (Santa Cruz Co., Ltd., TX, USA) at 4°C overnight, respectively, followed by washes with TBS-T and incubation with the peroxidase-conjugated secondary antibody at room temperature for 1 hour. The detections of labeling proteins were performed with enhanced-chemiluminescence western-blotting detections kits. And the relative protein levels were normalized to *β*-actin (Santa Cruz Co., Ltd.) protein as the internal standard.

### 2.12. Statistical Analysis

Data were presented as the mean ± SEM and statistical analyses were performed with Systat Sigma Plot software (version 12.00 for windows). Parametric data were analyzed by one-way ANOVA, followed by Tukey-Kramer test, and nonparametric data were analyzed by Kruskal-Wallis test, followed by Dunn's test. The mortality studies were analyzed by the Kaplan-Meier method. And *P* < 0.05 was considered to be statistically significant.

## 3. Results

### 3.1. The Effects of CFE on Survival Rates

**Figure 1:**
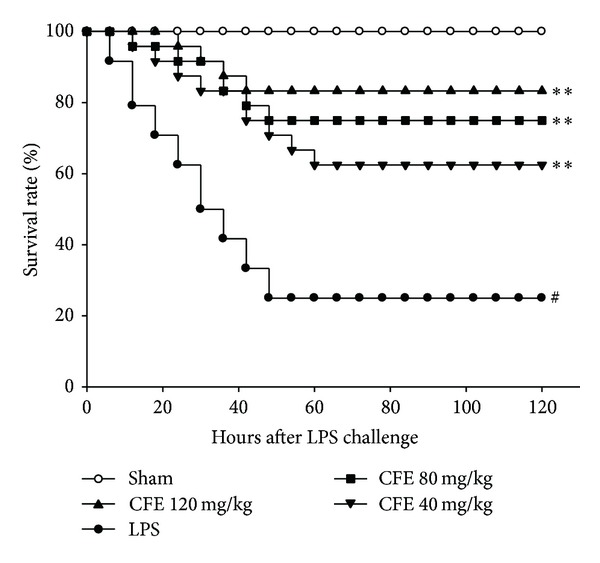
The effect of CFE on survival rates. Data was represented as the mean ± SEM (*n* = 24). ^#^ *P* < 0.01 compared to the sham group; *P < 0.05 and **P < 0.01 compared to the LPS group.

As shown in Figure 1, compared to the sham group with the survival rate of 100.0%, LPS challenge markedly declined the survival rate of the LPS group to about 25.0% within 120 hours 11 (*P* < 0.01). Conversely, survival rates of CFE-treated groups (40, 80, and 120 mg/kg) significantly increased to 62.5%, 75.0 %, and 83.3%, respectively, in a dose-dependent manner (for all, *P* < 0.01 versus the LPS group). Data indicated that CFE pretreatment possessed potential prevention against mortality in ALI mice induced by LPS.

### 3.2. The Effects of CFE on Lung W/D Ratio

**Figure 2:**
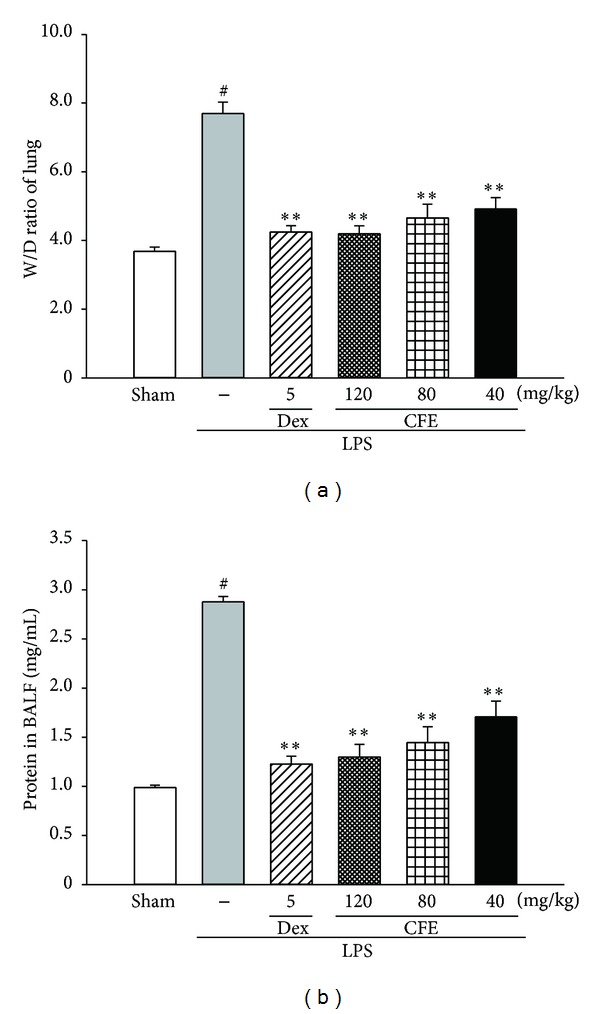
The effects of CFE on lung W/D ratio (a) and BALF protein content (b). Data was represented as the mean ± SEM (*n* = 10). ^#^ *P* < 0.01 compared to the sham group; *P < 0.05 and **P < 0.01 compared to the LPS group.

The W/D ratio of ALI mice was evaluated to assess the severity of pulmonary edema. And as shown in Figure 2(a), when compared with the sham group, there was a significant increase (approximately 2-fold) in the lung W/D ratio of the LPS group (*P* < 0.01). However, with the pretreatments of CFE (40, 80, and 120 mg/kg), the levels of lung W/D ratio were dose-dependently suppressed, as compared to the LPS group (for all, *P* < 0.01). Data showed that the lung W/D ratio was significantly suppressed by pretreatment with CFE.

### 3.3. The Effects of CFE on BALF Protein Content

The vascular permeability of the lung in mice was measured by the protein content of BALF. As compared to the sham group, LPS significantly boosted the BALF protein level of the LPS group (*P* < 0.01, Figure 2(b)). On the contrary, the protein contents of CFE groups (40, 80, and 120 mg/kg) were markedly suppressed in a dose-dependent manner, when compared with the LPS group (for all, *P* < 0.01).

### 3.4. The Effects of CFE on Cell Counts of BALF

**Figure 3:**
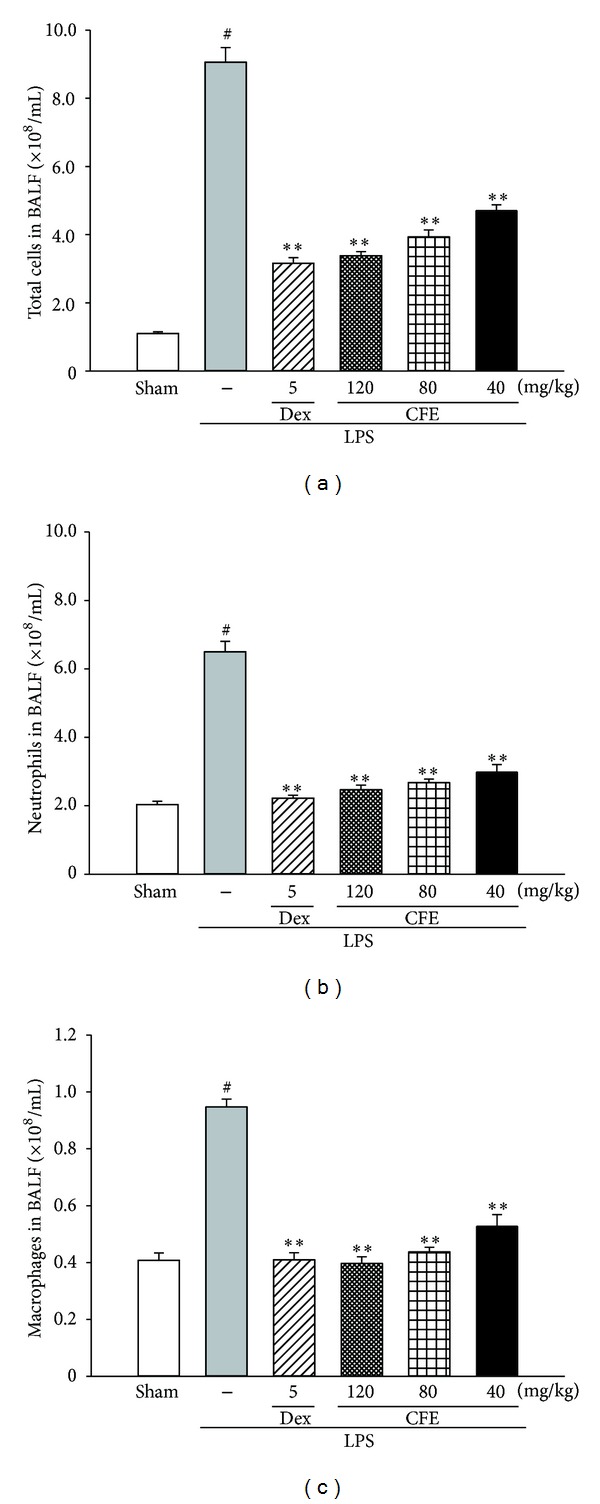
The effects of CFE on total cells (a), neutrophils (b), and macrophages (c) in BALF. Data was represented as the mean ± SEM (*n* = 10). ^#^ *P* < 0.01 compared to the sham group; *P < 0.05 and **P < 0.01 compared to the LPS group.

As shown in Figure 3, the BALF cell counts of LPS group demonstrated significant increases in the total cells (Figure 3(a)), neutrophils (Figure 3(b)), and macrophages (Figure 3(c)) (*P* < 0.01 versus the sham). However, pretreatments with CFE (40, 80, and 120 mg/kg) and Dex (5 mg/kg) markedly decreased all relevant cell counts in CFE groups and Dex group, respectively, (for all, *P* < 0.01 versus the LPS group).

### 3.5. The Effects of CFE on MPO Activity

**Figure 4:**
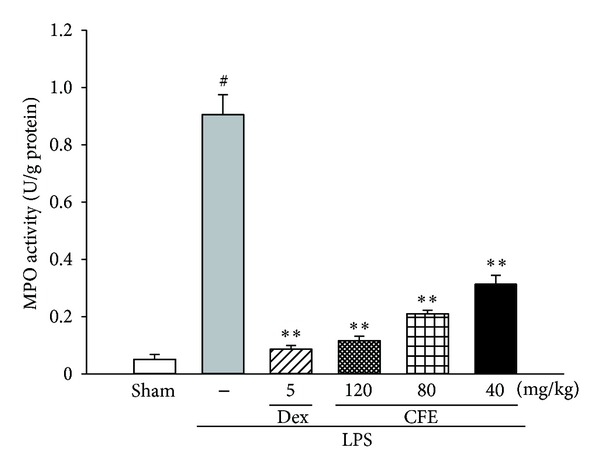
The effects of CFE on MPO activity. Data was represented as the mean ± SEM (*n* = 10). ^#^ *P* < 0.01 compared to the sham group; *P < 0.05 and **P < 0.01 compared to the LPS group.

MPO served as a functional index indicating neutrophils infiltration, which represented the levels of MPO-derived oxidants generation and lung tissue damage [5, 8, 14]. As expected outcomes of the LPS group, the MPO activity in lung tissue, was significantly elevated, about 9 folds of the sham group (*P* < 0.01, versus the sham group), which was shown in Figure 4. However, when compared to the LPS group, the MPO activities of the CFE-treated (40, 80, and 120 mg/kg) and Dex-treated (5 mg/kg) groups were significantly inhibited (for all, *P* < 0.01).

### 3.6. The Effects of CFE on Histopathological Examination

**Figure 5:**
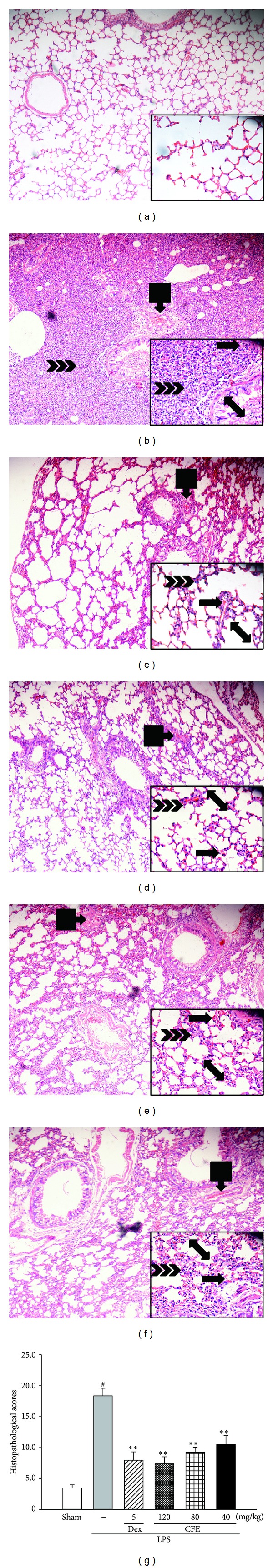
The effects of CFE on histopathological examination. Sham (a), LPS (b), LPS + 5 mg/kg Dex (c), LPS + 120 mg/kg CFE (d), LPS + 800 mg/kg CFE (e), LPS + 40 mg/kg CFE (f), and histologic changes scorings (G) (100x and 400x). Data was represented as the mean ± SEM (*n* = 10). ^#^ *P* < 0.01 compared to the sham group; *P < 0.05 and **P < 0.01 compared to the LPS group. Haemorrhage: 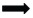; interstitial edema: 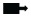; thickening of the alveolar wall: 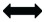; and infiltration of inflammatory cells: 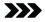.

Histopathological analyses were performed to investigate the effects of CFE on physiological parameters. As expected, the sham group displayed normal structures and no histopathological change in lung tissues (Figure 5(a)). On the other hand, with the challenge of LPS, the pulmonary function of LPS group was obviously impaired, with various histopathologic changes including haemorrhage, interstitial edema, thickening of the alveolar wall, and infiltration of inflammatory cells into the lung parenchyma and alveolar spaces (Figure 5(b)). As for experimental groups and positive group, histopathological changes were obviously abated by pretreatments of Dex (Figure 5(c)) and CFE (40 mg/kg, Figure 5(d); 80 mg/kg, Figure 5(e); and 120 mg/kg, Figure 5(f)), respectively, when compared to the LPS group. In addition, similarly inhibitory effects were found in semiquantitative assay by the histologic changes' scorings, which promoted the evaluation of severity of ALI (Figure 5(g)). Results demonstrated that pretreatment with CFE attenuated the severity of lungs injuries of ALI mice induced by LPS and improved the condition of lungs' tissues, in a dose-dependent manner.

### 3.7. The Effects of CFE on MDA, SOD, CAT, and GPx Levels

Oxidative stress plays vital important roles in the process of ALI induced by LPS, and oxidative damage induces lipid peroxidation of membrane phospholipids and inactivation of antioxidative enzymes (SOD, CAT, and GPx), while culminating in the MDA generation [5, 8, 14]. In the LPS group, MDA level in lung tissue was remarkably increased, compared to the sham group (*P* < 0.01), shown in Table 2. However, groups pretreated with CFE (40, 80, and 120 mg/kg) as well as Dex (5 mg/kg) showed significant declines in the MDA level (for all, *P* < 0.01 versus the LPS group). In addition, the activities of SOD, CAT, and GPx in LPS group were significantly abated, when compared with the sham group (for all, *P* < 0.01), as presented in Table 2. On the contrary, pretreatments with CFE (40, 80, and 120 mg/kg) significantly boosted the SOD, CAT, and GPx activities (for all, *P* < 0.01 versus the LPS group), in a dose-dependent manner.

### 3.8. The Effects of CFE on TNF-*α*, IL-1*β*, and IL-6 Levels

As compared to the sham group, the levels of TNF-*α*, IL-1*β*, and IL-6 in LPS group were remarkably raised (for all, *P* < 0.01, Table 3). Administration with CFE (40, 80, and 120 mg/kg) had inhibitory effects on the level of TNF-*α* (*P* < 0.05, versus the LPS group). In addition, pretreatment with CFE (40, 80, and 120 mg/kg) downregulated the levels of IL-1*β* and IL-6, as compared to the LPS group (for all, *P* < 0.05).

### 3.9. The Effects of CFE on TLR4/MyD88/NF-*κ*B Expressions

**Figure 6:**
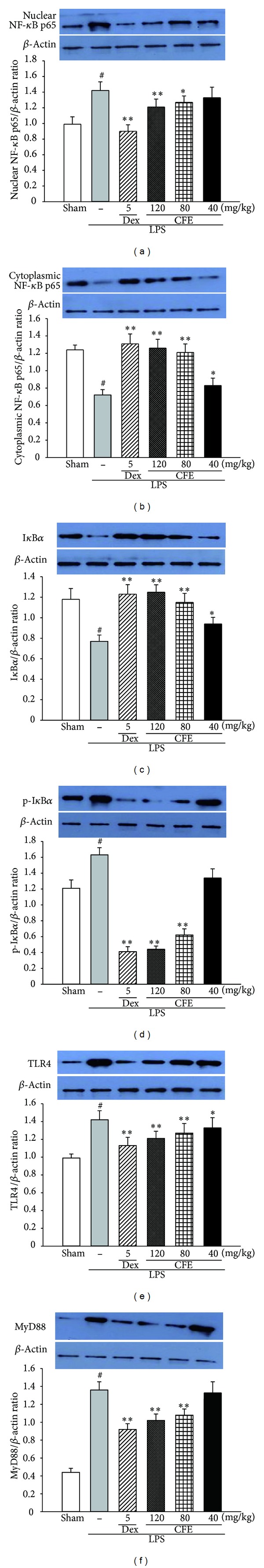
The effects of CFE on TLR4/MyD88/NF-*κ*B expressions. The levels of NF-*κ*B p65 in nucleus (a), NF-*κ*B p65 in nucleus cytoplasm (b), I*κ*B*α* (c), p-I*κ*B*α* (d), TLR4 (e), and MyD88 (f). Data was represented as the mean ± SEM (*n* = 10). ^#^ *P* < 0.01 compared to the sham group; *P < 0.05 and **P < 0.01 compared to the LPS group.

In order to probe the potential mechanisms of CFE in protection of LPS-induced ALI in mice, the expressions of TLR4, MyD88, and NF-*κ*B in lungs were further investigated. As shown in Figure 6, data of western-blot displayed that the expressions of TLR4/MyD88/NF-*κ*B signaling pathways were activated by LPS in the LPS group (for all, *P* < 0.01, versus the sham group). However, pretreatments with CFE (80 and 120 mg/kg) inhibited the phosphorylation of I*κ*B*α* and the expressions of TLR4 and MyD88 (for all, *P* < 0.05, versus the LPS group), despite the fact that the low dose of 40 mg/kg did not reduce the phosphorylation levels of I*κ*B*α* and MyD88, statistically. On the other hand, in CFE (80 and 120 mg/kg) groups, the expressions of p65 subunit NF-*κ*B in lungs were downregulated in nucleus and upregulated in cytoplasm, and the levels of I*κ*B*α* increased significantly (for all, *P* < 0.01, versus the LPS group), as shown in Figure 6. In this study, results showed that pretreatment with CFE (80 and 120 mg/kg) possessed simultaneous and efficient downregulations of TLR4 and MyD88-dependent NF-*κ*B signaling pathways, in LPS-induced ALI mice.

## 4. Discussions

In this present study, we evaluated the effects of CFE on LPS-induced ALI in mice. And data demonstrated that CFE abated LPS-induced lung histopathologic changes, declined the wet/dry ratio and proinflammatory cytokines productions, inhibited inflammatory cells migrations and protein leakages into the lungs, suppressed the levels of MPO and lipid peroxidation, upregulated the abilities of antioxidative enzyme, and downregulated the activations of NF-*κ*B and the expressions of TLR4 and MyD88. These suggested that CFE exerted potential protective effects against LPS-induced ALI.

LPS challenge leads to the leakages of serous fluids into lung tissues, resulting in the pulmonary oedema, a typical symptom of acute inflammatory responses in lung [22]. And pulmonary oedema is always evaluated via measuring a representative index, the lung W/D ratio [22, 23]. Based on the dose-dependent attenuation of the lung W/D ratio in CFE (40, 80, and 120 mg/kg) pretreated groups, pretreatment with CFE showed a significant inhibition of the pulmonary edema in ALI mice. On the other hand, the BALF total protein content, an index of lung permeability, was downregulated by pretreatment of CFE; it also demonstrated that pretreatment with CFE (40, 80, and 120 mg/kg) possessed attenuation action on lung permeability enhanced by LPS. LPS challenge also directly stimulates the infiltrations of inflammatory cells. Particularly, neutrophils migrating into the lung parenchyma and alveolar space have been indicated to have critical roles in the process of ALI [23, 24]. Neutrophils secrete MPO and lead to productions of MPO-derived oxidants and damage of lung tissues [23, 24]. Therefore the infiltration of neutrophils is usually represented by the ability of MPO [23, 24]. As expected, data showed that mice with LPS challenge presented massive infiltrations of inflammatory cells, including neutrophils and macrophages into the lung. However, pretreatment with CFE improved these changes by significantly decreasing the numbers of cells and abating LPS-induced increasing of MPO activity. These results also further proved that neutrophil respiratory burst and lung tissue damage were attenuated by CFE treatment. In addition, results of histopathologic examinations in lung tissues revealed that inflammatory responses and lung injuries in ALI mice were attenuated by CFE treatment in a dose-dependent manner. In summary, results of the alveolar-capillary barrier and inflammatory response as well as histological evidences demonstrated that CFE possessed significantly protective effects on LPS-induced ALI in mice.

Oxidative stress in lung tissue is also an important factor in the pathogenesis of ALI [5, 25, 26]; therefore, oxidative stress in the lung tissue was evaluated. Nowadays, the level of reactive oxygen species (ROS) is known as a classical index of oxidative stress [5, 26, 27]. ROS are chemically reactive molecules containing oxygen and are easy to react with biological macromolecules leading to lipid peroxidation, proteins inactivation, DNAs mutation, and finally tissue damage [5, 26, 27]. In the early stage of ALI induced by LPS, responses of neutrophils, including respiratory burst and degranulation, stimulate cells to rapidly release ROS, such as superoxide radicals (O_2_•^−^), hydrogen peroxide (H_2_O_2_), and hydroxyl radicals (OH•^−^) [25, 27, 28]. On the other hand, MPO is catalyzed to produce hypochlorous acid (HOCl) that is categorized as ROS, to respond for LPS challenge and/or kill other pathogens [25, 27, 28]. ROS primarily attack the polyunsaturated-fatty acids of cell and plasma membranes leading to the formations of MDA, a lipid peroxidation product [27, 29]. Thus, the accumulation of MDA is commonly used as a marker to manifest the degree of lipid peroxidation, to some extent, the level of oxidative stress and antioxidant status [26, 29]. With the results of MDA assay, pretreatment with CFE was found to have significant inhibitory effects on the formation of MDA, which clearly indicated that oxidative stress in lung tissues of ALI mice was alleviated. In addition, ROS have been proposed to mediate cell damage via a number of independent mechanisms including the inactivation of antioxidant defense systems consisting of a variety of antioxidant enzymes, such as SOD, CAT, and GPx [25, 29, 30]. These enzymes can minimize, scavenge, and eliminate the formation of ROS, protecting the host against oxidative stress-induced damage [30–32]. Hence, effective antioxidants can abate the oxidative stress of ALI by direct elimination of free radicals or by boosting defense systems of antioxidant enzymes [26, 30, 32, 33]. In this study, CFE also markedly upregulated the activities of SOD, CAT, and GPx, which were observed to be markedly decreased in sepsis-induced ALI rodents [34, 35] and in the ALI patients clinically [27, 36]. Therefore, combining with the results of MDA assay, we speculated that CFE could effectively reduce oxidative stress in ALI.

Clinical and experimental studies suggest that LPS induce the activation of alveolar macrophages and endothelial cells, which result in the productions of many proinflammatory and chemotactic cytokines [15, 37]. TNF-*α*, IL-1*β*, and IL-6 are well-characterized cytokines involved in the inflammatory responses of ALI [13, 38, 39]. These cytokines, combining with other proinflammatory factors, further stimulate neutrophils' infiltrations to migrate into lung tissues and initiate, amplify, and perpetuate the entire or focal inflammatory responses in ALI [15, 40]. TNF-*α*, mainly produced by monocytes/macrophages, is the earliest and primary endogenous mediator of the process of an inflammatory reaction and can elicit the inflammatory cascade, cause damage to the vascular endothelial cells, and induce alveolar epithelial cells to produce other cellular factors, such as IL-6 [40, 41]. Elevated TNF-*α* binds with a TNF-*α* acceptor in lung tissue, leading to the leakage of enzymes out of the organelle, which causes damage to the lung parenchyma [15]. IL-1*β* plays a key role in the progression of acute lung injury. It can inhibit fluid transportations across the distal lung epithelium to cause surfactant abnormalities and to increase protein permeability across the alveolar-capillary barrier [13]. IL-6 is one of the most common inflammatory cytokines, and its circulating levels have been shown to be excellent predictors of the severity of acute respiratory distress syndrome of different aetiologies, such as sepsis and acute pancreatitis [15, 40, 42]. In this present study, pretreatment with CFE significantly inhibited the production of TNF-*α*, IL-1*β*, and IL-6 in lung. The inhibition of proinflammatory factors productions was in accordance with the protective effects of CFE against histopathologic damage. And the suppression of proinflammatory cytokines by CFE treatment was supposed to contribute to its protective effects against ALI.

NF-*κ*B is an important nuclear transcription factor and plays a pivotal role in immune and inflammatory responses through the regulation of proinflammatory cytokines, chemokines, and adhesion molecules [12, 43, 44]. Uncontrolled activations of the NF-*κ*B pathways were involved in the pathogenesis of many acute and chronic inflammatory diseases, especially ALI [43, 45]. In normal conditions, NF-*κ*B is sequestered in the cytoplasm by I*κ*Bs [46, 47]. Once activated, NF-*κ*B p65 dissociates from its inhibitory proteins I*κ*B and translocates from the cytoplasm to the nucleus where it triggers the transcription of specific target genes such as TNF-*α*, IL-1*β*, and IL-6 [37, 48]. To detect the inhibitory mechanism of TNF-*α*, IL-1*β*, and IL-6 productions, we tested the effects of CFE on NF-*κ*B activation and I*κ*B degradation [37]. With the stimulation of LPS, the levels of phosphorylated I*κ*B protein and nucleus NF-*κ*B p65 protein were remarkably increased. However, this tendency was reversed by CFE pretreatment, as western-blot analysis showed that I*κ*B degradation and NF-*κ*B p65 activation were significantly blocked by pretreatment with CFE at the doses of 80 mg/kg and 120 mg/kg. In addition, we found that 40 mg/kg of CFE did not affect I*κ*B degradations and NF-*κ*B p65 activations statistically, in spite of the fact that it possessed inhibitory tendencies. Therefore, results suggested that the protective effects of CFE against LPS-induced ALI, to some extent, may be attributed to its roles in downregulation of NF-*κ*B pathways.

**Figure 7:**
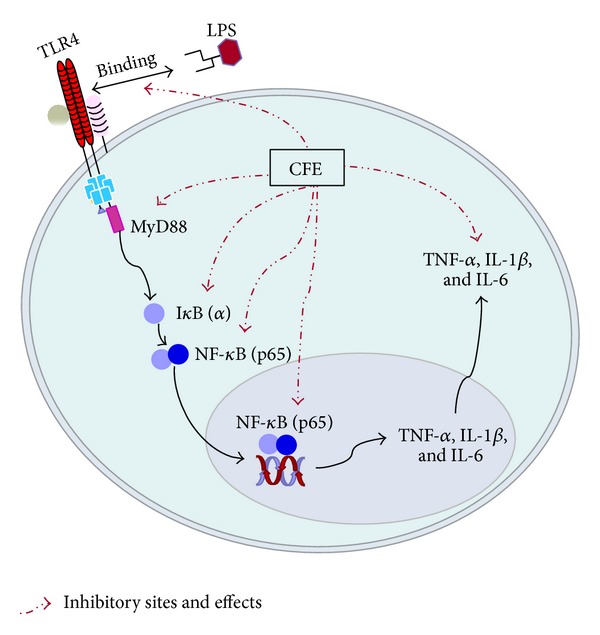
The underlying mechanism of CFE on the TLR4 signaling pathway. The red dotted arrows indicate the inhibitory sites and effects of CEF.

LPS play key roles in the development and progression of ALI [5, 22, 49, 50]. TLR4, serving as an important pattern recognition receptor of host immune responses and essential upstream sensor for LPS from pathogens and microorganisms, would detect LPS and then trigger the activation of NF-*κ*B and its downstream responses through MyD88 dependent or independent pathways [5, 7, 49, 50]. Thus TLR4 is the fundamental upstream sensor for LPS [7, 11, 49, 51]; it was necessary to probe whether the anti-inflammation action of CFE exerted though TLR4-mediated pathways. Our data showed that the enhanced expressions of TLR4 by LPS challenge were significantly downregulated with pretreatments of CFE at the doses of 80 and 120 mg/kg, which corresponded with the level changes of I*κ*B*α*, NF-*κ*B p65, and other proinflammatory cytokines, in ALI mice lung tissues. Therefore, in conclusion, as shown in Figure 7, we speculated that CFE could inhibit the binding of LPS to TLR4 in NF-*κ*B signaling pathways, leading to reductions of proinflammatory cytokines productions and attenuations of pulmonary inflammatory responses. However, without any further experiments to eliminate the involvement of MyD88-independent pathway, we could not address and demonstrate the conclusion that TLR4 activates NF-*κ*B via a MyD88-independent pathway. Therefore, explicit regulations of TLR4 signaling pathways by CFE require further studies.

## 5. Conclusions

The experimental evidence in this study demonstrated that CFE can effectively attenuate the LPS-induced ALI in mice. The protective effects of CFE were associated with the modulations of TLR4 signaling pathways. These experimental results suggested that CFE was a potential therapeutic drug for ALI.

## Figures and Tables

**Table 1:** The chemical profile of CFE.

Number	Components	Percentage (%)
1	Camphene	0.475^a^
2	*β*-Cymene	0.998^a^
3	Eucalyptol	3.091^a^
4	Linalool oxide	0.521^a^
5	*α*-Thujone	2.186^a^
6	*β*-Thujone	2.169^a^
7	Isothujol	1.094^a^
8	L-Pinocarveol	0.765^a^
9	d-Camphor	8.582^a^
10	*cis*-Verbenol	4.720^a^
11	*endo*-Borneol	7.845^a^
12	L-4-Terpineol	1.634^a^
13	*α*-Terpineol	1.022^a^
14	Myrtenol	1.054^a^
15	Cumaldehyde	0.486^a^
16	Bornyl acetate	2.948^a^
17	Thymol	3.071^a^
18	*β*-Caryophyllene	3.336^a^
19	*cis*-*β*-Farnesene	2.270^a^
20	*α*-Curcumene	5.932^a^
21	*δ*-Cadinene	1.815^a^
22	Spathulenol	1.362^a^
23	Caryophyllene oxide	8.460^a^
24	*γ*-Eudesmol	1.568^a^
25	T-Muurolol	1.487^a^
26	*α*-Gurjunene	2.161^a^
27	Aromadendrene	2.280^a^
28	*α*-Bisabolol	2.289^a^
29	Cubenol	1.742^a^
30	Longifolenaldehyde	2.572^a^
31	*α*-Bisabolol oxide	2.600^a^
32	Hexahydrofarnesyl acetone	1.212^a^
33	Ethyl hexadecanoate	1.362^a^
34	*α*-Linolenic acid	2.130^a^
35	Ethyl octadec-9,12-dienoate	2.470^a^
36	Chlorogenic acid	2.110^b^
37	Luteolin-7-glucoside	2.800^b^
38	Linarin	4.830^b^
39	Luteolin	1.140^b^
40	Acacetin	0.880^b^

^a^Identified by GC-MS analysis and the relative percentage calculated by integrated peak area in Agilent MSD Chemstation data analysis program.

^b^Identified and quantified by HPLC-PAD analysis, and the relative percentage was represented by the content quantitatively analyzed with peak areas under the standard curves.

**Table 2:** The effects of CFE on LPS-induced the levels of MDA, SOD, CAT, and GPx.

Groups	MDA (nmol/mg protein)	SOD (U/mg protein)	CAT (U/mg protein)	GPx (U/mg protein)
Sham	0.29 ± 0.02	1.03 ± 0.08	0.67 ± 0.05	0.32 ± 0.04
LPS	1.18 ± 0.10^#^	0.31 ± 0.02^#^	0.11 ± 0.03^#^	0.10 ± 0.03^#^
Dex (5 mg/kg)	0.62 ± 0.06∗∗	0.57 ± 0.06∗∗	0.39 ± 0.06∗∗	0.17 ± 0.03∗∗
CFE (120 mg/kg)	0.49 ± 0.06∗∗	0.62 ± 0.05∗∗	0.42 ± 0.03∗∗	0.20 ± 0.02∗∗
CFE (80 mg/kg)	0.53 ± 0.04∗∗	0.53 ± 0.05∗∗	0.38 ± 0.05∗∗	0.17 ± 0.05∗∗
CFE (40 mg/kg)	0.56 ± 0.06∗∗	0.44 ± 0.05∗∗	0.36 ± 0.06∗∗	0.16 ± 0.02∗∗

Data represented the mean ± SEM (*n* = 10). ^#^ *P* < 0.01 compared to the sham group; *P < 0.05 and **P < 0.01 compared to LPS group.

**Table 3:** The effects of CFE on LPS-induced the levels of TNF-*α*, IL-1*β*, and IL-6.

Groups	TNF-*α* (pg/mg protein)	IL-1*β* (ng/mg protein)	IL-6 (ng/mg protein)
Sham	31.97 ± 2.47	2.36 ± 0.22	5.52 ± 0.74
LPS	123.7 ± 4 9.36^#^	13. 41 ± 1.13^#^	20.20 ± 2.47^#^
Dex (5 mg/kg)	79.81 ± 13.56∗∗	5.28 ± 0.61∗∗	9.66 ± 1.57∗∗
CFE (120 mg/kg)	69.49 ± 9.71∗∗	5. 67 ± 0.83∗∗	10.80 ± 1.69∗∗
CFE (80 mg/kg)	77.43 ± 11.29∗∗	6.08 ± 0.52∗∗	11.96 ± 1.21∗∗
CFE (40 mg/kg)	93.49 ± 6.50∗	7. 92 ± 1.24∗	12.33 ± 1.09∗∗

Data represented the mean ± SEM (*n* = 10). ^#^ *P* < 0.01 compared to the sham group; *P < 0.05 and **P < 0.01 compared to LPS group.

## Abbreviations

CFE: The supercritical-carbon dioxide fluid extract of* Chrysanthemum indicum* Linné
LPS: Lipopolysaccharide
ALI: Acute lung injury
ARDS: Acute respiratory distress syndrome
MODS: Multiple organ dysfunction syndrome
BALF: Bronchoalveolar lavage fluid
ELISA: Enzyme linked immunosorbent assay
Dex: Dexamethasone
TNF-*α*: Tumor necrosis factor-*α*
IL-1*β*: Interleukin-1*β*
IL-6: Interleukin-6
MPO: Myeloperoxidase
MDA: Malondialdehyde
SOD: Superoxide dismutase
CAT: Catalase
GPx: Glutathione peroxidase
TLR4: Toll-like receptor 4
MyD88: Myeloid differentiation factor 88
NF-*κ*B p65: Nuclear factor kappa B p65
I*κ*B*α*: Inhibitory kappa B alpha
ROS: Reactive oxygen species
H&E: Hematoxylin and eosin.
